# Physical Work Demands of Maintenance Workers on Onshore Petroleum Facilities in Norway: An Observational Study Utilizing Wearable Sensor Technology

**DOI:** 10.1093/annweh/wxad022

**Published:** 2023-05-12

**Authors:** Svein O Tjøsvoll, Marius Steiro Fimland, Victor Gonzalez, Trine M Seeberg, Andreas Holtermann, Hilde Færevik, Øystein Wiggen

**Affiliations:** Department of Neuromedicine and Movement Science, Faculty of Medicine and Health Sciences, NTNU Norwegian University of Science and Technology, Edvard Griegs gate 8, Trondheim N-7491, Norway; Department of Neuromedicine and Movement Science, Faculty of Medicine and Health Sciences, NTNU Norwegian University of Science and Technology, Edvard Griegs gate 8, Trondheim N-7491, Norway; Unicare Helsefort Rehabilitation Centre, Rissa, Hysnesveien 11, 7112 Hasselvika, Norway; Department of Smart Sensor and Microsystems, SINTEF Digital, SINTEF AS, Dept. of Health Research, P.O. Box 124 Blindern, NO-0314 Oslo, Norway; Department of Neuromedicine and Movement Science, Faculty of Medicine and Health Sciences, NTNU Norwegian University of Science and Technology, Edvard Griegs gate 8, Trondheim N-7491, Norway; Department of Smart Sensor and Microsystems, SINTEF Digital, SINTEF AS, Dept. of Health Research, P.O. Box 124 Blindern, NO-0314 Oslo, Norway; Department of Musculoskeletal Disorders and Physical Workload, National Research Centre for the Working Environment, Lersø Parkallé 105, 2100 Copenhagen, Denmark; Department of Health Research, SINTEF Digital, SINTEF AS, P.O. Box 4760 Torgaarden, NO-7465 Trondheim, Norway; Department of Health Research, SINTEF Digital, SINTEF AS, P.O. Box 4760 Torgaarden, NO-7465 Trondheim, Norway

**Keywords:** accelerometry, ergonomics, human factors, manual labour, occupational health and safety, occupational physical activity, physical exposures, work-related physical activity

## Abstract

**Objectives:**

High physical work demands can cause musculoskeletal disorders and sick leave in petroleum workers. However, our knowledge of their physical work demands is scarce and based on self-report. The objective of our study is to work towards closing this knowledge gap by assessing the physical work demands of onshore petroleum maintenance workers using body-worn sensors.

**Methods:**

A total of 46 of 69 eligible maintenance workers (37 mechanics and 9 process technicians) from three onshore petroleum facilities in Norway filled in a questionnaire and diary and wore five accelerometers and a heart rate sensor for up to six consecutive workdays. Work-related physical activity and postures were classified using rule-based modelling in a modified version of the validated Acti4 software.

**Results:**

The onshore maintenance petroleum workers were working an average of 10 h a day and spent on average this time with 48% (SD = 16.5) sitting, 1% (SD = 2.8) lying down, 39% (SD = 16.2) in light physical activity, and 9% (SD = 3.8) in moderate to vigorous physical activity. During work hours while at feet, we found arm elevation ≥60° to be 11% (SD = 7.1) (68 min), and forward bending of the trunk ≥60° to be 2% (SD = 2.2) (14 min). The workers spent 2% (SD = 2.5) (12 minu) of the workhours kneeling. We observed a high inter-individual variation for all these work exposures. Moreover, 26% (12) of the workers conducted static standing for >30% of the workday, and 17% (8) spent more than half of the work hours >33% of their estimated maximal cardiovascular capacity.

**Conclusions:**

While onshore maintenance petroleum workers on average spend about half of the workday sitting or lying down, the remaining worktime is spent with a rather high duration of arm elevation and forward bending. Quite high fraction of the workers spends much of the workhours in static standing and kneeling. We see a substantial variation in these work exposures between the workers. The findings indicate a need for preventive measures in how work is organized and performed.

**What’s Important About This Paper?**

This study is important because it comprehensively measured the physical work demands among petroleum maintenance workers. The workdays involved rather long durations of arm elevation, forward bending and kneeling, and static standing; with high variation observed among workers. These findings indicate a need to modify how work is organized and performed to prevent musculoskeletal disorders and pain, injuries, disability pension, adverse cardiovascular events and sickness absence.

## Introduction

Workers in the petroleum industry are often exposed to challenging working environments. From 1992 to 2003, 47% of the total illness cases reported in the offshore petroleum industry in Norway were due to work-related musculoskeletal disorders (Morken *et al.*, 2007). Workers who had primarily maintenance tasks, such as mechanics and process technicians, represented most of the cases (40%). The most frequently self-reported regions of pain were the upper-extremities (53%), the lower back (20%), and the knee (12%) (Morken et al., 2007). According to the Petroleum Safety Authority Norway (2019), trends in ergonomic risks have remained stable throughout the last decade.

Mechanics and process technicians perform primarily maintenance tasks, such as troubleshooting, disassembly, installing, repairing machines and piping systems, design and construction of process facilities, changing, and adjusting various components on gas tanks, pumps, and valves (Morken *et al.*, 2007, Merkus *et al.*, 2015). These work tasks require manual material handling involving prolonged standing on hard surfaces, pushing, pulling, carrying, and heavy lifting of equipment, often in repetitive, monotonous movement patterns and awkward body positions (Morken *et al.*, 2007). These are all risk factors for musculoskeletal disorders, sickness absence, disability pensions, and early retirement (Morken *et al.*, 2004, Morken *et al.*, 2007, Andersen *et al.*, 2016, van der Molen *et al.*, 2017, Lunde *et al.*, 2019, Wang *et al.*, 2020, Hulshof *et al.*, 2021a, Hulshof *et al.*, 2021b). Additionally, a study found that 39% of mechanics and 34% of process technicians reported by questionnaires to have a high physical workload (Morken *et al.*, 2007).

Worldwide, only a few studies have utilized device-worn measurements of physical work demands in occupations with typical industrial work, such as construction and manufacturing (Koch *et al.*, 2017, Jørgensen *et al.*, 2019, Lunde *et al.*, 2019, Merkus *et al.*, 2019, Lunde *et al.*, 2021). These studies used 2–4 four body-worn sensors on the thigh, hip, upper back, and upper arm and measured from 3 to 6 consecutive days. Only one study included heart rate measurements (Merkus *et al.*, 2019). To our knowledge, all data on work demands in petroleum workers have been collected via self-reported measurements, which are imprecise and can be prone to recall bias (Koch *et al.*, 2016). Device-worn measurements are less constrained by these limitations and advances in microelectromechanical technology allow accurate body-worn solutions for measuring ergonomic risk factors (Korshoj *et al.*, 2014, Skotte *et al.*, 2014, Hallman *et al.*, 2015, Stemland *et al.*, 2015, Hendriksen *et al.*, 2020, Tjøsvoll *et al.*, 2022a).

There is, therefore, a need to fill the knowledge gap of the physical work demands for maintenance workers in the petroleum industry using precise device-worn measurements. Hence, the purpose of this study was to assess the physical work demands of mechanics and process technicians on onshore petroleum facilities in Norway using body-worn accelerometers and heart rate monitors.

## Materials and methods

### Study population

Maintenance workers consisting of mechanics and process technicians normally working 8-h dayshifts with a minimum of 50% full-time equivalent employment (minimum 20 working hours a week) were recruited from three of eight onshore petroleum facilities in Norway. All workers were provided written and oral information about the research project and gave written consent prior to the study. Inclusion criteria included that the primary work tasks had to be manual material handling. Exclusion criteria were: (i) office work, (ii) night work, (iii) physical disability not allowing normal behaviour, (iv) pregnancy, and (v) bandage, band aid, or adhesive allergies. The study was conducted according to the Declaration of Helsinki and approved by the Regional Committees for Medical Research Ethics, Central Norway (No.: 190964). We have previously investigated the physical work demands of home care workers and the description of the methods and results are partly overlapping (Tjøsvoll *et al.*, 2022b).

### Data collection

Data were collected from questionnaires, anthropometric measurements, cardiorespiratory fitness tests, and device-worn measurements from October 2021 to April 2022. The data were stored and analysed in accordance with current data protection guidelines (GDPR.EU, 2021). All workers completed a questionnaire regarding socio-demography, health-related-, and workplace factors (NTNU, 2021).

### Anthropometrics

A digital body scale and a wall-mounted measuring tape (SECA 206, SECA Medical Measuring Systems and Scales, Birmingham, UK) were used for baseline measurements of height and weight.

### Sensor measurements

Technical devices can pose a risk of ignition under certain operating conditions in petroleum facilities. Thus, risk assessment of the devices utilized in this research project was carried out, and a temporary approval for use of equipment at the given locations was given.

Five triaxial AX3 accelerometers (Axivity Ltd., Newcastle upon Tyne, UK) were attached to the skin of participants, using double-sided adhesive tape (3M; Witre, Halden, Norway) and sealed with waterproof medical tape (Opsite Flexifix; Mediq, Oslo, Norway). The sensors were worn 24 h each day for a period of up to six consecutive workdays with a sampling frequency of 25 Hz and a range of ± 8G. The accelerometers were mounted: (i) below the head of the fibula, on the proximal and lateral position of the calf for classification of kneeling, (ii) on the distal, anterior, and medial position of the femur (approximately 10 cm above the superior crest of the patella), (iii) approximately 10 cm below the iliac crest of the hip for classification of lying, sitting, moving, walking, running, stair climbing, and running, (iv) on the upper back at the level of Th1–Th2 vertebrae for classification of forward bending of the trunk, and (v) on the dominant upper arm, approximately at the insertion (tuberositas deltoidea) of the deltoid muscle for classification of arm elevation (Skotte *et al.*, 2014, Korshoj *et al.*, 2014, Hallman *et al.*, 2015). After sensor placement, the participants were instructed to perform a calibration procedure that required standing still and jumping and were asked to do this calibration procedure every morning. Participants were provided a paper diary to record the daily activity of: (i) when they woke up in the morning, (ii) sensor calibration jump, (iii) when they arrived at work, (iv) when they finished work, and (v) when they went to sleep.

Firstbeat Bodyguard 2 monitor (Firstbeat Technologies Ltd., Jyväskylä, Finland) (Parak *et al.*, 2015) was used for the assessment of heart rate and worn up to six consecutive days, detecting the beat-to-beat intervals with a sampling frequency of 1000 Hz. Electrocardiography electrodes that were single-use and pre-gelled (Arbo H92SG) were mounted on the thorax of the onshore petroleum workers. This sensor had to be removed by the participants before water activities and then reattached.

### Aerobic workload

Applying the following formula, the aerobic workload was calculated as the percent heart rate reserve (HRR) (Karvonen *et al.*, 1957):


$$\begin{array}{l} \%HRR\;=\;\frac{HR_{HRwork}-HR_{HR\min}\;}{HR_{HR\max\;}-HR_{HR\min}}\;\times 100\%, \end{array}$$

_$HR_{min}$_ was calculated using a moving window over an average of 10 beats for the lowest total heartbeats every night, throughout the measurement period of each worker. $HR_{max}$ was determined using the following formula (Tanaka *et al.*, 2001):


$$\begin{array}{l} HR_{\max}=208-0.7\;\times Age \end{array}$$

Maximum %HRR was calculated from the average of the highest measured heart rate over all workdays. Average %HRR was calculated from the total values from all workdays.

### Assessment of cardiorespiratory fitness

The submaximal Ekblom–Bak test (Björkman *et al.*, 2016, GIH, 2021) was used to estimate the maximal aerobic capacity ($\dot{V}O_{2max}$) and conducted on the cycle ergometer model Monark 939E (Monark AB, Varberg, Sweden). A polar H10 or Garmin HRM-dual heart rate sensor belt was used to record the heart rate.

### Data processing

The software OMGUI (version 1.0.0.43; Axivity Ltd., Newcastle upon Tyne, UK) was used for configuration of the AX3 accelerometers, and a modified version of the custom-made MATLAB software Acti4 (Stemland *et al.*, 2015) was used for further processing of the sensor data.

Applying rule-based models, the software is capable of classifying activities and postures, such as lying, sitting, standing, walking slowly, walking fast, moving (neither standing still nor walking), running, cycling, stair climbing, arm elevation, forward bending of the trunk and kneeling with high sensitivity and specificity (≥95%) (Korshoj *et al.*, 2014; Skotte *et al.*, 2014; Hallman *et al.*, 2015, Hendriksen *et al.*, 2020, Tjøsvoll *et al.*, 2022a). Non-wear time was classified when no movement was detected in non-sleep periods for intervals of more than one and a half hours. Firstbeat Uploader software (Firstbeat Technologies Ltd., Jyväskylä, Finland) with default settings was used for downloading heart rate data and then processed together with the accelerometer data using the Acti4 software. Average work-related physical activities, postures, and HRR for each worker were calculated by adding the respective values from all valid workdays for each worker. Errors ≥50% in the heart rate data were removed from the dataset.

Activity diaries for each worker were analysed with the Acti4 software and divided into the following categories: (i) sensor calibration, (ii) working hours, (iii) after working hours, and (iv) sleep. After processing all data, a batch analysis was conducted in the Acti4 software and imported to a CSV file. Working hours from the dataset were derived using Python (version 3.10.0; Python Software Foundation 2001–2021). At least two workdays with ≥4 working hours each day were required from each worker to be eligible for further analysis (Skotte *et al.*, 2014, Jørgensen *et al.*, 2019). Due to a one-week window for data collection in each onshore facility, sensors were mounted on Monday or on Wednesday and removed after work on Friday. While workers were typically off-duty on weekends, some workers worked during the weekend. Therefore, we have measurements between two and six days. Arm elevation data from three workers and forward trunk inclination data from two workers were removed because of technical errors.

### Statistics

Descriptive statistics were calculated for all participants, that is, weighted mean, standard deviations, and percentages. Statistical processing and analysis of the data were carried out in Python with custom-made scripts.

## Results

**Figure 1.**
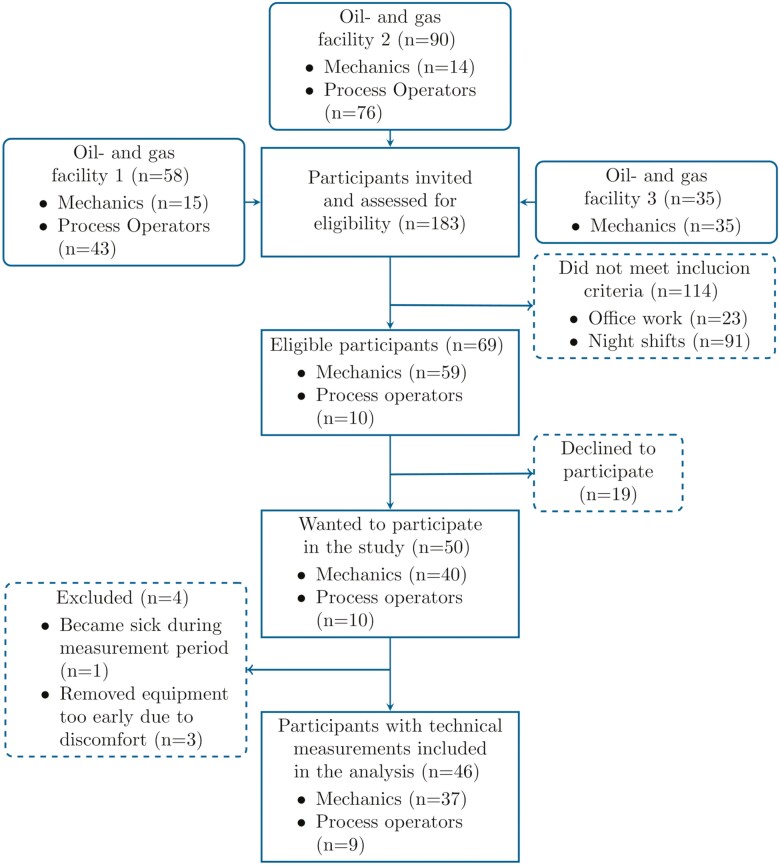
Flow of participants.

We recorded 1751 h of accelerometer data and 1688 h of heart rate data over an average of 4 workdays on 46 onshore maintenance petroleum workers. The participant flow can be seen in Fig. 1. Concisely, 69 of 183 onshore maintenance petroleum workers were eligible for participation in this observational study. All the included onshore maintenance petroleum workers had 100% employment in the three included onshore petroleum facilities. We measured a mean of 9.9 (SD = 2.4) working hours a day as several participants worked overtime during the measurement period. Demographics, health, and work characteristics of the 46 participants who completed the study are depicted in Table 1.

**Table 1.** Demographics, health, and work characteristics of onshore maintenance petroleum workers (*n* = 46).

Demographic characteristics	*N* (%)	Mean (SD)^a^
Age (years)	46 (100)	29.8 (11.1)
Gender	46 (100)	
Female	8 (17.4)	
Male	38 (82.6)	
Cardiorespiratory fitness^b^ (ml/kg/min)	46 (100)	
Female	8 (17.4)	36.9 (6.4)
Male	38 (84.3)	44.5 (8)
Body mass index (kg/m^2^)	46 (100)	
Female	8 (17.4)	25.5 (4.2)
Male	38 (84.3)	27.3 (4.5)
Marital status	41 (89.2)	
Married/partner	21 (45.7)	
Not married/living alone	20 (43.5)	
Education (years)	46 (100)	
High school (up to 3 years)	13 (28.3)	
Certificate of completed apprenticeship	29 (63)	
College/university	4 (8.7)	
Worked in Petroleum industry (years)	46 (100)	6 (6.9)
Work demands^c^	46 (100)	
Work requires you to work fast	46 (100)	2.5 (0.7)
Work requires you to work hard	46 (100)	2.7 (0.5)
Work requires too much effort	46 (100)	2.5 (0.7)
Work requires ingenuity	46 (100)	3 (0.6)
Decide how to perform your work tasks	46 (100)	3.2 (0.8)
Decide your own work tasks	46 (100)	2.6 (0.6)
Tired after work	46 (100)	2.1 (0.5)
WAI^d^	46 (100)	8.8 (1.4)
Perceived health^e^	46 (100)	3.1 (0.4)
Experienced pain at least three consecutive months during the last year	46 (100)	
Yes	26 (56.5)	
No	20 (43.5)	
Regions with pain at least three consecutive months during the last year	10 (21.7)	
Neck	8 (17.4)	
Shoulders	10 (21.7)	
Elbow	2 (4.3)	
Wrist and fingers	4 (8.7)	
Upper back	3 (6.5)	
Lower back	10 (21.7)	
Hip	1 (2.2)	
Knees	9 (19.6)	
Calves	1 (2.2)	
Feet and ankles	5 (10.9)	
Prevented activities during work because of this pain	3 (6.5)	
Sick leave last 12 months	26 (56.5)	
<2 weeks	23 (50)	
>2 weeks	3 (6.5)	
Self-reported leisure-time physical activity	46 (100)	
Never	6 (13)	
Once a week	11 (23.9)	
2–3 times a week	17 (37)	
Every day	12 (26)	

^a^SD = standard deviation.

^b^ $\dot{V}O_{2max}$ .

^c^1 = Never, 2 = No/rarely, 3 = Yes/sometimes 4 = Yes/frequently.

^d^Work ability index, 0 = Cannot work, 10 = Best.

^e^1 = Poor, 2 = Not that good, 3 = Good, 4 = Very good

**Figure 2.**
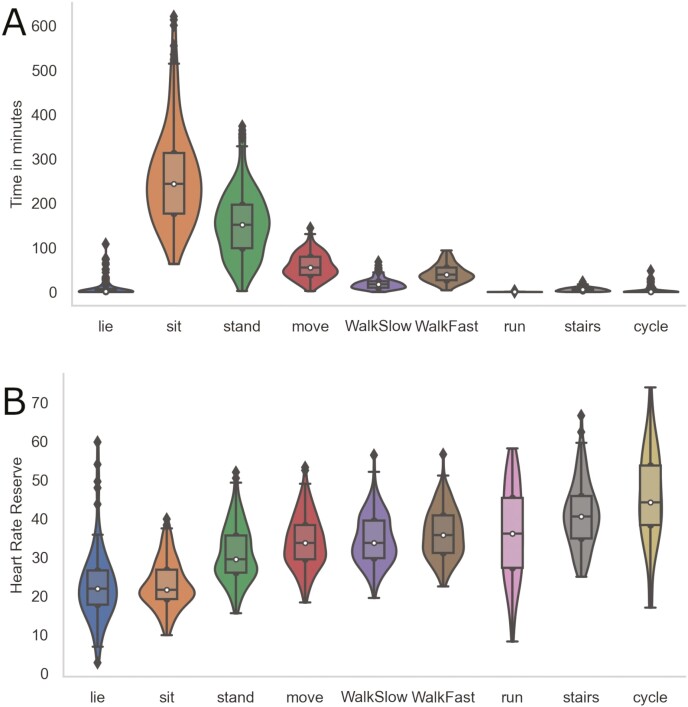
Time in minutes during work for 46 onshore maintenance petroleum workers spent in work-related physical activity (A) and the corresponding percent heart rate reserve (B) using device-based measurements. The violin plots depict information about the distribution of the data. The box shows the median, 25th and 75th percentile, and the black lines indicate the rest of the distribution.

Fig. 2 shows the distribution of the average time spent in minutes and percent in work-related physical activity and the relation between HRR and work-related physical activity. Table 2 depicts the weighted mean time spent in percent and minutes in work-related physical activities, demanding postures at work, weighted mean %HRR and SDs. The composition was as follows: sitting was the most common physical behaviour (48%), a small portion of the day was spent lying down (1.3%), and the remaining time was in work-related physical activity (48%). The subdivision of the latter category was: 26% static standing, 9.8% moving, 3.2% walking slowly, 7.1% walking fast, 0.03% running, 1% stair climbing, and 0.7% cycling (0.7%). The average %HRR was highest for cycling (45.6%), stair climbing (40.9%), running (36.2%), and walking fast (36.2%). The lowest %HRR was measured for sitting (23%) and lying down (23%). The average %HRR for the remaining work-related physical activities were static standing (30.9%), moving (34%), and walking slowly (34.5%).

**Table 2:** Weighted mean of physical work demands of 46 onshore maintenance petroleum workers during working hours of all workdays.

Work-related physical activity	Time in %	SD	Time in minutes	SD	Mean %HRR	SD
Lying	1.3	2.8	8.2	16.8	23.1	9.4
Sitting	48.2	16.5	283.2	115.4	23	6
Static standing	26	12.1	157.8	80.5	30.9	7.2
Moving	9.8	4.4	60.1	29.2	34	6.8
Walking slowly	3.2	2	19.5	13	34.5	6.4
Walking fast	7.1	3.1	43.7	21.7	36.2	6.3
Running	0.0	0.1	0.2	0.3	36.2	11.8
Stair climbing	1	0.7	5.9	4.4	40.9	8.3
Cycling	0.7	1.4	3.3	7.1	45.6	12
Light intensity physical activity	39	16.2	237.4	109.5	32	6.9
Moderate to vigorous physical activity	8.8	3.8	53.1	26.1	39.8	7.2
Total physical activity	47.8	17.8	290.5	124.5	35.1	7
Demanding postures	Time in %	SD	Time in minutes	SD	–	–
Kneeling	1.8	2.5	11.5	15.1	–	–
Arm elevation ≥30°	50.1	12.2	294.8	106.7	–	–
Arm elevation while on feet ≥30°	43.2	12.6	253.7	102.6	–	–
Arm elevation ≥60°	12.8	7.3	76.6	52.6	–	–
Arm elevation while on feet ≥60°	11.3	7.1	67.9	51.6	–	–
Arm elevation ≥90°	2.2	2.3	12.9	11.5	–	–
Arm elevation while on feet ≥90°	1.8	2.2	10.9	10.8	–	–
Forward trunk inclination ≥30°	19	11.8	113.5	76.8	–	–
Forward trunk inclination while on feet ≥30°	7.5	6.2	46.9	40.9	–	–
Forward trunk inclination ≥60°	3.9	6	21.0	20.3	–	–
Forward trunk inclination while on feet ≥60°	2.2	2.2	13.6	12.6	–	–
Forward trunk inclination ≥90°	0.6	0.8	3.9	5	–	–
Forward trunk inclination while on feet ≥90°	0.5	0.6	2.9	4	–	–

Values are means and standard deviations (SD). N: number; %HRR: heart rate reserve; LIPA: light intensity physical activity (static standing, moving, walking slowly); MVPA: moderate to vigorous physical activity (walking fast, running, stair climbing, and cycling); total activity: LIPA and MVPA combined.

**Figure 3.**
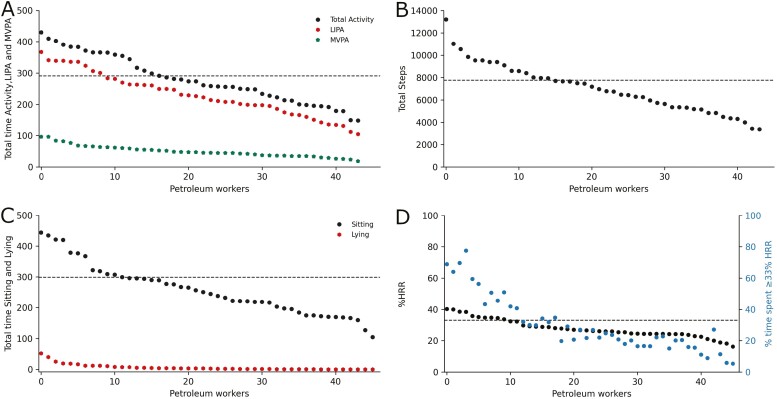
Weighted mean of work-related exposures for each of the 46 onshore maintenance petroleum workers during the workday. Individual participants are depicted as dots during working hours on the *x* axis and on the *y* axis (A) minutes in LIPA (light intensity physical activity: static standing, moving, and walking slowly) and MVPA (moderate to vigorous physical activity: walking fast, running, stair climbing, and cycling) and total activity (LIPA + MVPA); (B) Total steps; (C) total minutes sitting and lying down; and (D) mean %HRR (heart rate reserve) and percentage time spent with at least 33% HRR. The black horizontal dashed lines in A–C depict the threshold for 50% of the workday and in D, the threshold for 33% HRR.

**Figure 4.**
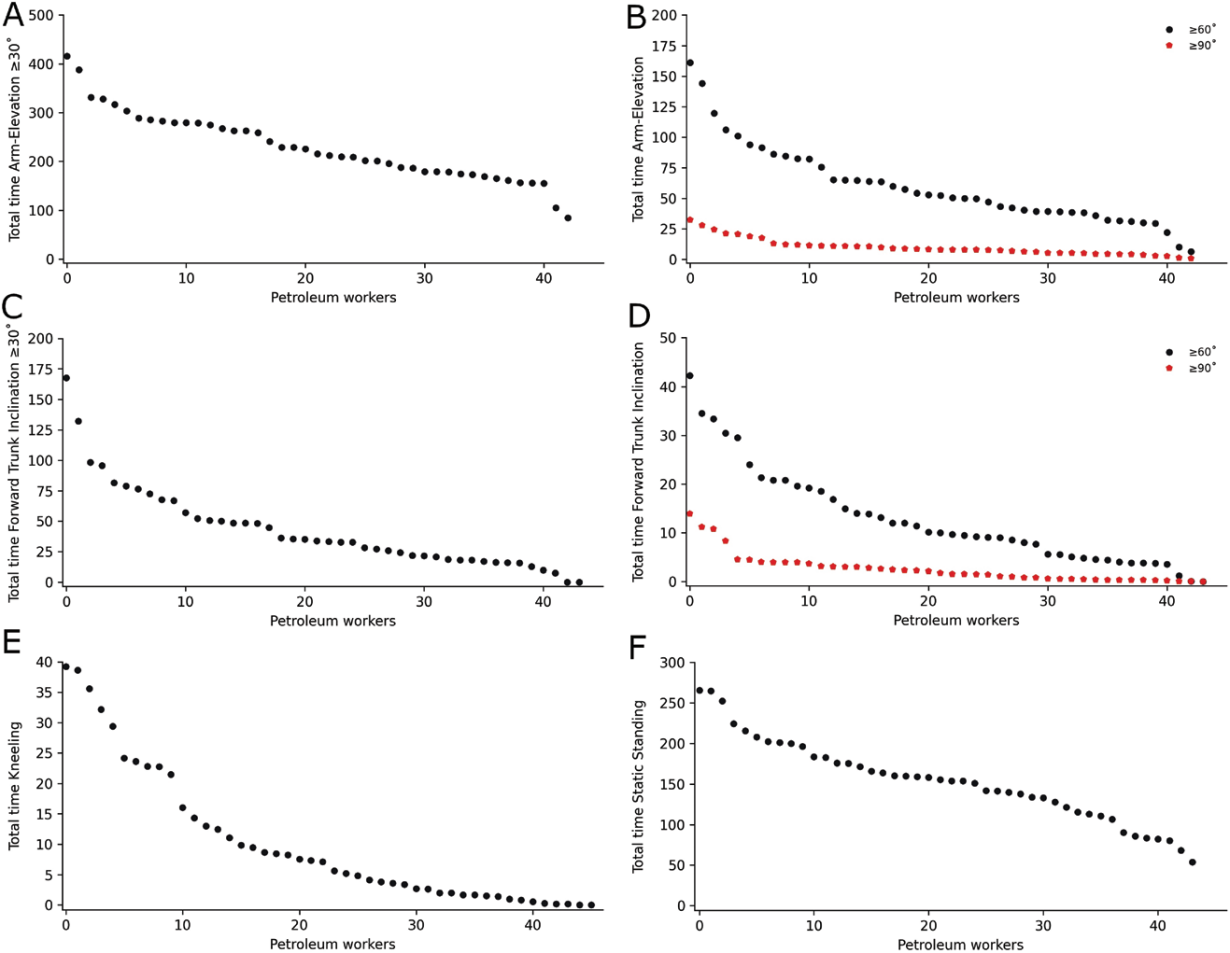
Weighted mean of demanding postures for each 46 onshore maintenance petroleum workers during the workday. Individual participants are depicted as dots on the *x* axis and on the *y* axis (A) total minutes of arm elevation ≥30° while on feet, (B) total minutes of ≥60° and ≥90° arm elevation while on feet, (C) total minutes ≥30° of forward bending of the trunk while on feet, (D) total minutes ≥60° and ≥90° of forward bending of the trunk while on feet, (E) total minutes kneeling and (F) total minutes static standing.

Figs. 3 and 4 depict the weighted mean of work-related physical activity, cardiovascular workload, and demanding postures respectively, on an individual level during the workday.

## Discussion

This is the first study to comprehensively assess physical work demands among onshore maintenance petroleum workers using device-worn measurements. We found that mechanics and process technicians on average spent about half the workday sitting, while they were statically standing 26% and performing various work-related physical activities 22% (walking slowly, walking fast, moving, running, stair climbing, cycling) of the worktime. The fraction of workhours spent statically standing and kneeling, and with arm elevation and forward bending of the trunk was rather high. The cardiorespiratory aerobic workload was measured to be within the recommended ‘safe limits’. Notably, there was an extensive uneven distribution of physical work demands among workers, meaning that some workers were exposed to much higher physical work demands than others.

In contrast to the well-documented benefits of leisure-time physical activity, large prospective cohort studies report that high amounts of work-related walking and lifting are risk factors for adverse cardiovascular events (Holtermann *et al.*, 2021), sickness absence (Gupta *et al.*, 2020), and disability pension due to musculoskeletal disorders (Fimland *et al.*, 2018). On average, the onshore maintenance petroleum workers spent about 48% of the workday on their feet, which at a glance could be interpreted as a seemingly balanced level of work-related physical activity. However, a closer inspection of the individual data revealed a rather high inter-individual variation of work-related physical activity. Actually, more than one third (16) of the workers spent 50–73% (5–7 h) of the workday on their feet. An increasing body of evidence suggests that exposure to high levels of work-related physical activity can cause persistent fatigue and elevation of the resting blood pressure, resulting in a hemodynamic imbalance (Clays *et al.*, 2012). Another proposed mechanism for adverse health effects due to high work-related physical activity, is the accumulation of inflammatory cytokines, inducing alterations of the endothelial function, eventually increasing the risk of atherosclerosis (Wang *et al.*, 2016). Consequently, our findings indicate that a substantial fraction of onshore maintenance petroleum workers might be exposed to harmful amounts of work-related physical activities.

The average time spent static standing was 26% (about 2.6 h), with a considerable inter-individual variability (Fig. 4F). As much as 26% (12) of the workers were statically standing more than 30% (about 3 h) of the workday. Prolonged static standing is associated with the pooling of blood in the veins, increased blood pressure, lower back-, knee-, and hip pain (Waters and Dick, 2015, Coenen *et al.*, 2018). Currently available occupational health and safety guidance on static standing is though based on limited high-quality evidence due to mostly being derived from self-reported measurements and laboratory studies (Coenen *et al.*, 2018). However, a report by the European Agency for Safety and Health at Work proposed that workers subjected to prolonged constrained standing should strive for a composition of 60% sitting, 30% standing and 10% moving/walking/cycling throughout the workday (EU-OSHA, 2021). Considering this recommendation, our results indicate that more than one-fourth of the workers could be at risk of adverse health effects from prolonged static standing. Moreover, it could be inferred that onshore maintenance petroleum workers should generally sit more and move less. Additionally, some studies have found that increased sitting time could be beneficial for blue-collar workers, reducing risk of low back pain due to increased time for rest and recovery (Lunde *et al.*, 2017, Korshøj *et al.*, 2018).

On average, onshore maintenance petroleum workers were well within the proposed safe threshold for cardiovascular workload. A proposed upper safe limit by the International Labor Organization for whole-body work is that workers on average, should be within one third of their maximal aerobic capacity during an 8-h workday (Bonjer, 1971), and more recent studies suggest to stay within an average of 30% HRR for 10-h work shifts (Jørgensen, 1985, Rodgers, 1986, Wu and Wang, 2002, Brighenti-Zogg *et al.*, 2016). Despite the average %HRR showing an acceptable level of cardiovascular workload, our results revealed that 22% (10) of the onshore maintenance petroleum workers were subjected to levels above one third of their estimated maximal aerobic capacity. Moreover, 17% (8) spent 51–78% of the workday above this threshold, indicating that these workers are exposed to high durations of cardiovascular workloads throughout the workday. Hence, some workers could be at risk of increased musculoskeletal pain and other detrimental health effects, especially those with low physical capacity (Väisänen *et al.*, 2021, Merkus *et al.*, 2022). Furthermore, sitting and lying down are frequently referred to as sedentary behaviours, but this does not always imply absolute rest in the workplace as shown by an average HRR of 23%. Documentation work and control room operations are common work tasks performed using active upper body work in a seated position in onshore petroleum facilities. Furthermore, we found that some workers had ≥30% HRR while sitting and lying down as shown in Fig. 2B, implying that several maintenance tasks were likely performed while sitting or lying. Moreover, psychosocial stress might also have contributed to elevate the HRR in various postures (Taelman *et al.*, 2009).

Regarding demanding postures, we found that on average, onshore maintenance petroleum workers were conducting arm elevation while on feet ≥30° for 43% (254 min), ≥60° for 11.3% (68 min), and ≥90° for 1.8% (11 min) during working hours. Moreover, a high inter-individual variation was found. The Danish Dphacto cohort used the same methods to assess physical work demands in various blue-collar professions, where manufacturing was perhaps the most comparable to the workers in the present study. Danish manufacturers were exposed to ≥60° of 6.3% and ≥90° of 1.3% arm elevation while on feet (Jørgensen *et al.*, 2019), which was lower than we observed in onshore maintenance petroleum workers. According to multiple evidence syntheses, work-related arm elevation is associated with the development of shoulder disorders (Wærsted *et al.*, 2020, Seidler *et al.*, 2020). Moreover, a recent study by (Gupta *et al.*, 2021) investigating the dose–response relationship between arm elevation and prospective long-term sickness absence using the same device-based measurements as in our present study, found that arm elevation while on feet ≥30° for ≥124 min, ≥60° for ≥37 min and ≥90° for ≥8 min was associated with a twofold increased risk of long-term sickness absence. The inter-individual variation found in our study (Fig. 4A,B), showed that 89% (41), 74% (34), and 37% (17) of the workers, on average, conducted elevation of the upper extremities while on their feet ≥30°, ≥60°, and ≥90° at or above these durations. Hence, our results indicate that these workers could be at risk of long-term sickness absence. A suggested pathophysiological mechanism is that high frequency and pressure of the muscle and tendons and other surrounding soft tissue, that is, capsule, ligaments, and bursa elicit fatigue due to prolonged activation of the muscles, restricted microvasculature blood flow, eventually causing tendinopathy or rotator-cuff lesions (van der Molen *et al.*, 2017, Seidler *et al.*, 2020).

We found that the average levels of exposure to work-related forward bending of the trunk while on feet ≥30° was 7.5% (47 min) and ≥60° was 2.2% (14 min) with a high inter-individual variation. Onshore maintenance petroleum workers were conducting less work-related forward bending of the trunk than manufacturers (≥60° of 3%) in the Danish Dphacto cohort (Jørgensen *et al.*, 2019). It has been hypothesized that acute or cumulative impact of work-related forward bending of the trunk may lead to endplate microfractures, increased intradiscal pressure, causing protrusion and degeneration of the intervertebral disc (Coenen *et al.*, 2013). Work-related forward bending of the trunk in combination with awkward and heavy lifting has been found to amplify the risk of low back pain (Veiersted, 2017). Although we have no information on the context in which work-related forward bending of the trunk occurred, we can assume that there were instances of awkward and heavy lifting as this is common in industrial work (Sterud and Tynes, 2013, Sterud *et al.*, 2014). A recent 4-year prospective study using device-based measurements found that work-related forward bending of the trunk while on feet >30° for 40 min and >60° for 10 min was associated with 12.2% and 12.3% increased absolute risk of long-term sickness absence in blue-collar workers (Gupta *et al.*, 2022). Our results showed (Fig. 4C,D,d) that 39% (18) and 48% (22) of the workers were above these durations. Thus, a high portion of the workers could have an increased risk of long-term sickness absence.

Onshore maintenance petroleum workers were on average conducting 1.8% (12 min) of work-related kneeling. Additionally, we found a high inter-individual variation with some workers exposed to durations of kneeling for up to 40 min during working hours. There were only comparable data of device-worn measurements of kneeling from Danish childcare workers (2.5%) (Holtermann *et al.*, 2020) and Norwegian home care workers (0.8%) (Tjøsvoll *et al.*, 2022b). Onshore maintenance petroleum workers were on average having a higher exposure to kneeling than Norwegian home care workers, but lower levels than childcare in Denmark. Although the average level of exposure to kneeling was lower for onshore maintenance petroleum workers than Danish childcare workers, there has been found a higher risk for knee disorders in industry-based studies (Wang *et al.*, 2020). Cumulative mechanical load causing degeneration of the cartilage has been purposed as a plausible pathophysiological mechanism for work-related knee disorders (Wang *et al.*, 2020). Furthermore, evidence suggests that work-related kneeling throughout days, weeks, months, and years, increases the risk of degenerative knee disorders such as osteoarthritis but also meniscal tear, tendinopathy, and pain (Morken *et al.*, 2007, Verbeek *et al.*, 2017, Wang *et al.*, 2020, Hulshof *et al.*, 2021a). Currently, available guidance for a safe upper limit for exposure to kneeling is based on self-reports and is inconsistent. Despite there is presently no objectively measured data to support that a certain amount of kneeling could impose detrimental health effects, we cannot rule out that workers exposed to the highest durations of kneeling have an increased risk of adverse health effects.

Predominant preventive strategies to reduce musculoskeletal pain has been by implementing ergonomic aids to reduce loads, or by organizing work so that it is varied and do not cause harm (NorwegianWorkingEnvironmentAct, 2022). Despite these efforts, there are still massive challenges related to work-related musculoskeletal disorders and pain. The recently proposed “Goldilocks Work” approach, proposed by Holtermann *et al.* (2019) may be useful. This concept study proposed a 4-step procedure to make work health promoting. Precise knowledge about the workers’ physical work demands, health status is essential information before specifying a goal for improvement (e.g., improved musculoskeletal health) and reorganizing or modifying work tasks to reach that goal (Holtermann *et al.*, 2019). The comprehensive information provided in the current study provides a good starting point for investigating interventions that can make work health promoting for onshore maintenance petroleum workers, for example, by redistributing physical work demands, enabling more workers getting a “just right” amount of work-related physical activity. A recent study found that it was feasible to modify productive work towards a “just right” level of work-related physical activities, showing tendencies of reduction in perceived pain, fatigue, and improved energy levels of industrial workers (Lerche *et al.*, 2021).

A limitation of the current observational study was that only 67% of the eligible onshore maintenance petroleum workers completed the study. Thus, we cannot eliminate the possibility of selection bias, as workers not willing to participate could have different demographics, for example, higher age and poorer health. Due to a limited time window of one week of data collection in each onshore petroleum facility, we only had access to process technicians assigned to these dayshifts; therefore, the number of available process technicians was lower than for mechanics as fewer process technicians were working day shifts. In addition, we had only access to three of eight onshore petroleum facilities in Norway. As sensors were only mounted on one side of the body, it is possible that the amount of kneeling was underestimated (Tjøsvoll *et al.*, 2022a). There is also some uncertainty about the individual values for work intensity (HRR) as maximum heart rate is estimated and not measured directly through a maximal test, as this was not feasible in the current work setting. Finally, a general limitation relates to the technical measurements currently available in field-based sensor technology, which is not able to capture the level of muscle torque exerted due to handling external loads.

## Conclusion

This study helps to fill a knowledge gap on the physical work demands of onshore maintenance petroleum workers by using device-worn measurements. While onshore maintenance petroleum workers on average spend about half of the workday sitting or lying down, the remaining worktime is spent with a rather high duration of arm elevation, forward bending and kneeling. Quite high fraction of the workers spend much of the workhours in static standing and kneeling. We see a substantial variation in these work exposures between the workers. The findings indicate a need for preventive measures in how work is organized and performed. We encourage future research to use this knowledge to investigate health-promoting measures for onshore maintenance petroleum workers.

## Data Availability

Collected data and research protocol are available from the corresponding author on reasonable request.
